# IgG Responses to the Plasmodium falciparum Antigen VAR2CSA in Colombia Are Restricted to Pregnancy and Are Not Induced by Exposure to Plasmodium vivax

**DOI:** 10.1128/IAI.00136-18

**Published:** 2018-07-23

**Authors:** Mary Lopez-Perez, Mads Delbo Larsen, Rafael Bayarri-Olmos, Paulina Ampomah, Liz Stevenson, Myriam Arévalo-Herrera, Sócrates Herrera, Lars Hviid

**Affiliations:** a Centre for Medical Parasitology, Department of Immunology and Microbiology, Faculty of Health and Medical Sciences, University of Copenhagen, Copenhagen, Denmark; b Centre for Medical Parasitology, Department of Infectious Diseases, Rigshospitalet, Copenhagen, Denmark; c Laboratory of Molecular Medicine, Department of Clinical Immunology, Rigshospitalet, Copenhagen, Denmark; d Department of Biomedical Sciences, School of Allied Health Sciences, University of Cape Coast, Cape Coast, Ghana; e Caucaseco Scientific Research Center, Cali, Colombia; f Faculty of Health, Universidad del Valle, Cali, Colombia; University of South Florida

**Keywords:** antibodies, baculovirus, CHO cells, Colombia, insect cells, malaria, PfEMP1, Plasmodium falciparum, Plasmodium vivax, pregnancy, PvDBP, VAR2CSA, IgG, adaptive immunity, humoral immunity, placental malaria, recombinant antigens

## Abstract

Clinical immunity to malaria is associated with the acquisition of IgG specific for members of the Plasmodium falciparum erythrocyte membrane protein 1 (PfEMP1) family of clonally variant antigens on the surface of infected erythrocytes (IEs). The VAR2CSA subtype of PfEMP1 mediates IE binding in the placenta. VAR2CSA-specific IgG is normally acquired only after exposure to placental parasites. However, it was recently reported that men and children from Colombia often have high levels of functional VAR2CSA-specific IgG. This potentially undermines the current understanding of malaria immunity in pregnant women, and we thus conducted a study to assess further the levels of VAR2CSA-specific IgG in pregnant and nonpregnant Colombians. Plasma IgG against two full-length recombinant PfEMP1 proteins (one of the VAR2CSA type and one not) produced in baculovirus-transfected insect cells was detected frequently among Colombian men, children, and pregnant women with acute or previous malaria exposure. In contrast, IgG reactivity to a homologous full-length VAR2CSA-type protein expressed in Chinese hamster ovary (CHO) cells was low and infrequent among the Colombian plasma samples, as was reactivity to both corresponding native PfEMP1 proteins. Moreover, human and rabbit antibodies specific for Plasmodium vivax Duffy-binding protein (PvDBP), a protein with some homology to PfEMP1, did not react with VAR2CSA-type recombinant or native proteins, although the mouse monoclonal and PvDBP-specific antibody 3D10 was weakly reactive with recombinant proteins expressed in baculovirus-transfected insect cells. Our data indicate that the previously reported Colombian IgG reactivity to recombinant VAR2CSA is not malaria specific and that the acquisition of VAR2CSA-specific IgG is restricted to pregnancy, in Colombia and elsewhere.

## Abstract (precis)

Clinical immunity to malaria is associated with the acquisition of IgG specific for members of the Plasmodium falciparum erythrocyte membrane protein 1 (PfEMP1) family of clonally variant antigens on the surface of infected erythrocytes (IEs). The VAR2CSA subtype of PfEMP1 mediates IE binding in the placenta.

## INTRODUCTION

Malaria remains an important public health problem in large parts of the world. An estimated 216 million malaria cases and 445,000 malaria-related deaths occurred worldwide in 2016, most of them caused by Plasmodium falciparum in Africa (1). Outside Africa, Plasmodium vivax is responsible for about one-third of all malaria cases, including most cases in Latin America (1). Colombia, with approximately 8 million people living in areas where malaria is endemic, ranks third in the American continent in terms of malaria transmission, with half a million cases being reported between 2007 and 2013 (1, 2).

In areas with stable P. falciparum transmission, children and pregnant women are at a particularly high risk of malaria. This is because substantial protective immunity is acquired during childhood and adolescence, making clinical episodes and severe cases uncommon among adults (3). Malaria protection is mainly antibody mediated, and antigens like P. falciparum erythrocyte membrane protein 1 (PfEMP1) expressed on the infected erythrocyte (IE) surface are important targets (4). When women become pregnant, particularly for the first time, they become highly susceptible to malaria, despite any clinical immunity acquired earlier in life (5). This appears related to the ability of P. falciparum parasites to express a particular PfEMP1 subtype called VAR2CSA, which is antigenically distinct from all other PfEMP1 proteins and facilitates the selective accumulation of IEs in the placenta (6, 7). The expression of VAR2CSA is generally assumed to be incompatible with parasite survival in nonpregnant individuals (6, 8). Therefore, the acquisition of VAR2CSA-specific IgG is normally regarded as being pregnancy restricted, despite a few reports of sporadic, low levels of VAR2CSA-specific IgG among P. falciparum-exposed men, children, and nulligravidae (9–12).

The intensity of P. falciparum transmission in Colombia is low. Although at least 1 million women of reproductive age live in areas of the country where malaria is endemic, malaria in pregnancy, including placental malaria, is uncommon (13–15). It was therefore highly surprising when Gnidehou et al. reported a high prevalence of VAR2CSA-specific IgG in Colombia, not only in women with a history of pregnancy but also among nulligravidae, men, and children living in areas of the country where malaria is endemic (16). That same group recently proposed that the high VAR2CSA reactivity among Colombians might be related to cross-reactive antibodies induced by the P. vivax Duffy-binding protein (PvDBP) (17), which has low-level homology to PfEMP1.

The above-described findings from Colombia either point to a completely new and unanticipated mode of acquisition of VAR2CSA-specific IgG or suggest that the current understanding of immunity to placental malaria is incomplete. We therefore set out to shed additional light on the prevalence and specificity of IgG recognizing VAR2CSA-type PfEMP1 in Colombian populations.

## RESULTS

### IgG recognizing recombinant PfEMP1 proteins expressed in baculovirus-transfected insect cells is prevalent in plasma from Colombian pregnant women, men, and children.

**FIG 1:**
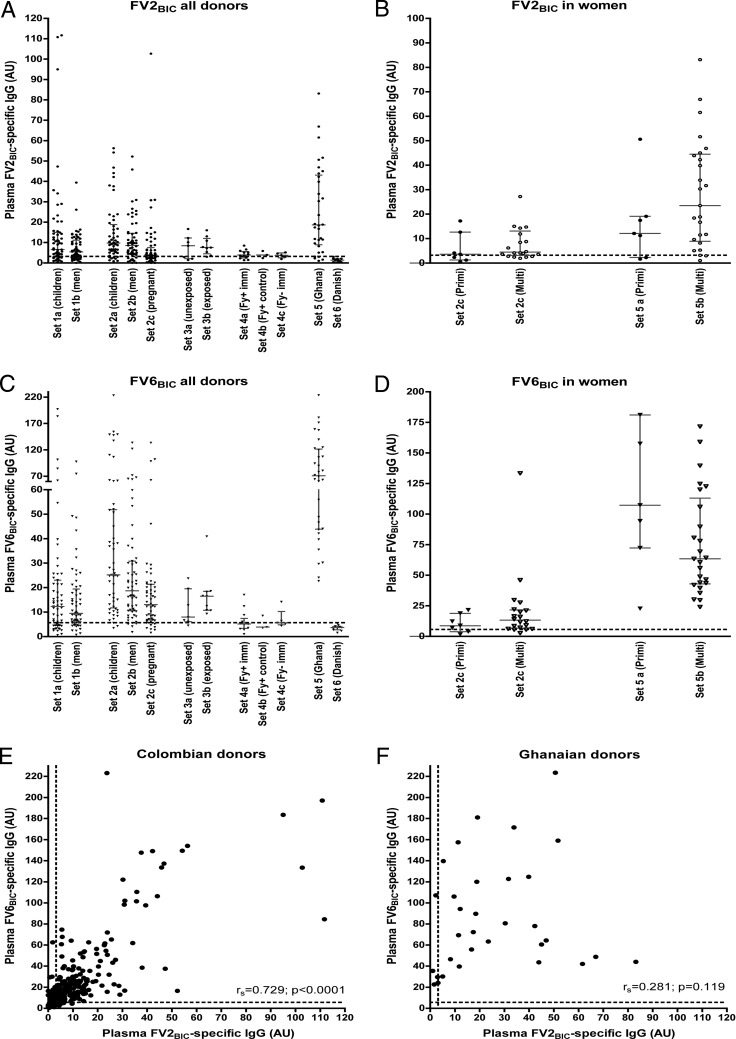
Antibody response to full-length recombinant PfEMP1 proteins produced in baculovirus-transfected insect cells. (A to D) IgG levels against a recombinant, full-length VAR2CSA-type PfEMP1 protein (FV2_BIC_) (A and B) and a full-length non-VAR2CSA-type PfEMP1 protein (FV6_BIC_) (C and D) produced in baculovirus-transfected insect cells were determined by an ELISA. Individual data for all samples sets are described in Table 1 (A and C). Individual data for samples in set 2c (Colombian pregnant women) and set 5 (Ghanaian women) were sorted by parity (primigravid [Primi] and multigravid [Multi]) (B and D). Medians and interquartile ranges are also shown. (E and F) Correlation (*r_s_*) between IgG levels specific for FV2_BIC_ and FV6_BIC_ in Colombian (set 1 and set 2) (E) and Ghanaian (set 5) (F) samples. The statistical significance levels of the correlations are also shown. In all panels, the values are expressed in arbitrary units (AU) (see Materials and Methods for details), and dashed lines indicate the negative cutoff values (mean levels plus 2 SD for Danish controls).

Significant plasma levels of IgG specific for VAR2CSA-type PfEMP1 are usually restricted to P. falciparum-exposed women who are or have recently been pregnant, and the levels generally correlate with parity among such women (18, 19). However, Gnidehou et al. recently reported that this does not appear to be the case in Colombia, as antibodies to recombinant VAR2CSA proteins did not depend on parity and were equally prevalent among malaria-exposed women, men, and children (16). To assess this surprising finding, we first measured levels of FV2_BIC_-specific IgG in plasma samples from different groups of Colombian donors (Fig. 1A). All the samples listed in Table 1 were included in these enzyme-linked immunosorbent assays (ELISAs). In agreement with data from the previous Colombian study, FV2_BIC_-specific IgG was detected frequently in samples from all the malaria-exposed donor groups studied here (set 1, set 2, and set 3b). With the exception of exposed men (set 1b; *P* < 0.001) and pregnant women (set 2c; *P* < 0.001), the levels were not statistically significantly different from those observed in samples from nonpregnant Ghanaian women who had been pregnant one or more times previously (set 5). Plasma levels of FV2_BIC_-specific IgG were significantly lower among unexposed Colombians (set 3a, set 4a, and set 4b; enrollment samples) than among exposed Colombians (set 1 and set 2; *P* < 0.001) but were still significantly higher than the levels among negative-control donors from Denmark (set 6; *P* < 0.001) (Fig. 1A). As reported previously by Gnidehou et al. (16), levels among the pregnant Colombian women studied did not differ between primigravidae and multigravidae (Fig. 1B). Although the FV2_BIC_-specific IgG levels also did not differ significantly between primigravidae and multigravidae among the Ghanaian women included here, such a difference was evident in the much larger sample set (18) from which the samples in set 5 were randomly drawn.

**TABLE 1:** General characteristics of samples and donors used

Sample set	Subset	No. of samples (no. of samples tested by flow cytometry)^*a*^	Median age (yr) (range)	No. of individuals infected by P. falciparum/no. of individuals infected by P. vivax^*b*^	Parasitemia (geometric mean no. of parasites/μl) (95% confidence interval)^*c*^	Median no. of previous episodes (interquartile range)^*d*^	Reference(s)^*e*^
1 (exposed, healthy)	Children	59 (10)	12 (2–16)			2 (1–3)	50
	Men	61 (7)	44 (19–80)			3 (2–5)	50
2 (naturally infected)	Children	53 (18)	13 (4–16)	22/31	4,122 (3,163–5,371)	2 (1–4)	15, 51
	Men	59 (19)	34 (18–70)	28/31	3,457 (2,352–5,082)	4 (2–7)	15, 51
	Pregnant women	56 (56)	21 (11–43)	77/23	3,980 (2,698–5,872)	2 (1–3)	51, 52
3 (PvCHMI)	Unexposed	7 (7)^*f*^	31 (22–38)				25
	Exposed	9 (0)	28 (19–37)				25
4 (PvRAS trial)	Fy^+^ (immunized)	12 (12)^*f*^	29 (21–40)				27
	Fy^+^ (controls)	3 (3)^*f*^	39 (27–41)				27
	Fy^−^ (immunized)	5 (0)	24 (19–37)				27
5 (Ghanaian women)	Primigravidae	7 (7)		0/0		Not available	18
	Multigravidae	25 (25)		0/0		Not available	18
6 (Danish adults)	Unexposed	11 (11)		0/0	0	0	

a Shown are numbers of samples in each set, and those with the highest ELISA reactivity to FV2_BIC_ by an ELISA and tested by flow cytometry are indicated in parentheses.

b There were no mixed infections.

c Parasites detected in Giemsa-stained thin smears.

d Median numbers (interquartile ranges) of self-reported malaria episodes during the individuals' lifetime.

e Original study reports.

f Only the enrollment samples were tested by flow cytometry.

We also measured plasma levels of IgG specific for another full-length recombinant protein (FV6_BIC_), expressed as FV2_BIC_ but representing a PfEMP1 antigen that is commonly recognized by plasma IgG from naturally exposed African donors. The expression of this PfEMP1 antigen is not restricted to parasites infecting pregnant women (18). The results (Fig. 1C and D) were strikingly similar to those obtained with FV2_BIC_. Furthermore, there was a significant and strong positive correlation between the FV2_BIC_ and FV6_BIC_ data among the Colombian samples (Fig. 1E) that was not observed in the corresponding data from Ghana (Fig. 1F). Taken together, these findings are in agreement with data from the previous study by Gnidehou et al. (16) and extend the surprisingly high and prevalent plasma antibody reactivity to recombinant VAR2CSA-type PfEMP1 among Colombian donors to comprise reactivity to a recombinant PfEMP1 protein (FV6_BIC_) that is not restricted to pregnancy (18, 20).

### IgG recognizing native VAR2CSA-type PfEMP1 expressed on the surface of P. falciparum-infected erythrocytes is rare in plasma from Colombian individuals.

**FIG 2:**
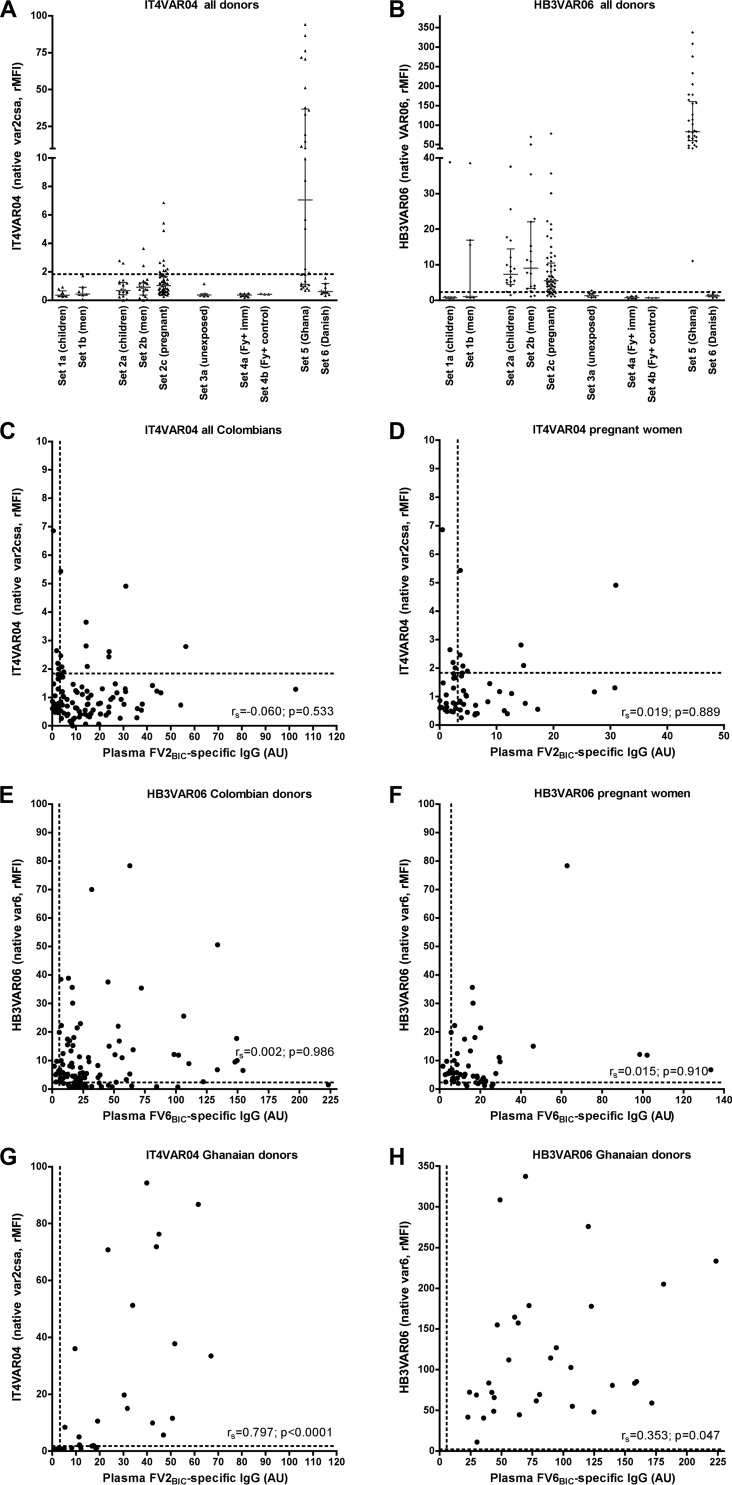
Antibody responses to native PfEMP1 and recombinant PfEMP1 proteins produced in baculovirus-transfected insect cells. (A and B) Levels of IgG specific for native VAR2CSA-type (IT4VAR04) PfEMP1 (A) and native non-VAR2CSA-type (HB3VAR06) PfEMP1 (B) expressed on the surface of IEs and measured by flow cytometry. (C and D) Correlation (*r_s_*) between IgG levels specific for recombinant (FV2_BIC_) and native (IT4VAR04) VAR2CSA-type PfEMP1 in samples from Colombian exposed, healthy, and naturally infected individuals (set 1 and set 2) (C) or from Colombian pregnant women only (D). (E and F) Correlation (*r_s_*) between IgG levels specific for recombinant (FV6_BIC_) and native (HB3VAR06) non-VAR2CSA-type PfEMP1 in Colombian samples (set 1 and set 2) (E) or in samples from Colombian pregnant women only (set 2c) (F). (G and H) Correlation (*r_s_*) between IgG levels specific for recombinant (FV2_BIC_) and native (IT4VAR04) VAR2CSA-type PfEMP1 (G) and between IgG levels specific for recombinant (FV6_BIC_) and native (HB3VAR06) non-VAR2CSA-type PfEMP1 (H) in samples from Ghanaian women. The statistical significance levels of the correlations are also shown. The panel layout is the same as in Fig. 1.

To substantiate the above-described findings, we next measured plasma IgG reactivity against erythrocytes infected with P. falciparum parasites selected *in vitro* to express the PfEMP1 antigens represented by the recombinant proteins used in the ELISAs described above. Subsets of the plasma samples in the various sets, selected to include those with the highest FV2_BIC_ reactivity by ELISAs, were used, as specified in Table 1. Between 7% (children and men) and 20% (pregnant women) of the samples from Colombia had IgG levels to IT4VAR04 (the native counterpart of FV2) above the negative cutoff level (Fig. 2A). Sixty-three percent of the nonpregnant Ghanaian women had levels of IT4VAR04-specific IgG above the cutoff. Between 61% (children) and 84% (pregnant women) of samples had levels of IgG recognizing IEs expressing HB3VAR06 (the native counterpart of FV6) above the cutoff (Fig. 2B). Antibody levels to the corresponding native and recombinant antigens did not correlate significantly, regardless of whether all the Colombian samples (Fig. 2C and E) or only the samples from the Colombian pregnant women (Fig. 2D and F) were considered. In contrast, plasma from Ghanaian women with a pregnancy history reacted strongly with both IT4VAR04 and HB3VAR06 (Fig. 2A and B; see also Fig. S1A and S2A in the supplemental material). Furthermore, levels of IgG recognizing the corresponding native and recombinant versions of either antigen were positively and significantly correlated among the samples from the Ghanaian women (Fig. 2G and H). These results suggest that the plasma samples from the Colombian donors recognized a component(s) that is present in both recombinant PfEMP1 antigens expressed in baculovirus-transfected insect cells but is not present in the corresponding native antigens and therefore is possibly not related to malaria exposure.

### IgG recognizing recombinant VAR2CSA-type PfEMP1 expressed in CHO cells is rare in plasma from nonpregnant Colombian individuals.

**FIG 3:**
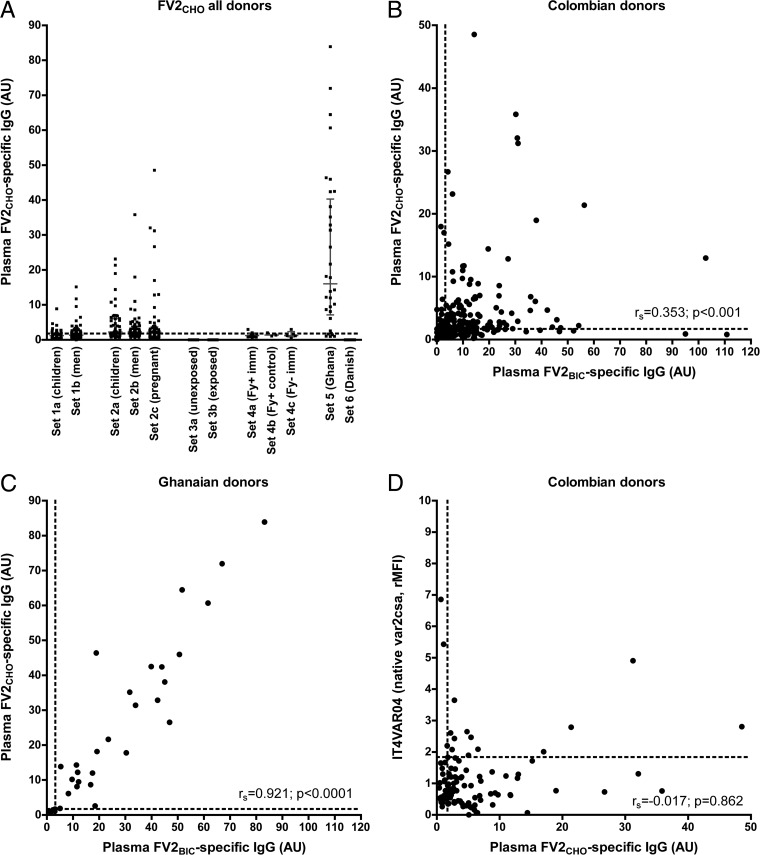
Antibody responses to native PfEMP1 and recombinant PfEMP1 proteins produced in insect or CHO cells. IgG levels against recombinant (FV2_BIC_ and FV2_CHO_) and the corresponding native (IT4VAR04) VAR2CSA-type PfEMP1 proteins were determined by an ELISA (recombinant proteins) or flow cytometry (native protein). (A) Individual FV2_CHO_-specific IgG levels in all samples (sample sets 1 to 6). (B and C) Correlation (*r_s_*) between IgG levels specific for FV2_BIC_ and FV2_CHO_ in Colombian (set 1 and set 2) (B) and Ghanaian (set 5) (C) samples. (D) Correlation (*r_s_*) between IgG levels specific for FV2_CHO_ and the corresponding native VAR2CSA-type protein in Colombian samples (set 1 and set 2). The panel layout is the same as in Fig. 1.

To assess the hypothesis that the reactivity to FV2_BIC_ among the Colombian donors was not P. falciparum specific, we generated another recombinant full-length PfEMP1 antigen containing the same amino acid sequence as that of FV2_BIC_ but expressed in a mammalian cell line (Chinese hamster ovary [CHO]) rather than in insect cells. Levels of IgG to this protein (FV2_CHO_) were generally low among all groups of Colombian donors (Fig. 3A). The differences between the Colombian and Ghanaian donors were statistically significant for all the groups except sets 2a and 2b, despite the fact that the Ghanaian women were not pregnant and therefore were not expected to have high levels of VAR2CSA-specific IgG, due to the rapid decline of these antibodies postpartum (21, 22). FV2_CHO_-specific IgG levels were weakly and positively correlated with IgG levels to FV2_BIC_ (Fig. 3B), in contrast to the plasma samples from the Ghanaian donors, where IgG levels to the these antigens were very strongly correlated (Fig. 3C). Antibody levels to FV2_CHO_ and the corresponding native protein did not correlate significantly when all the samples from Colombia were considered (Fig. 3D). When the samples were further analyzed according to donor type (see Fig. S3 in the supplemental material), a weak positive correlation between FV2_CHO_- and IT4VAR04-specific IgG was observed in the samples from the Colombian pregnant women (set 2c) but not in the samples from children (set 1a and set 2a) or men (set 1b and set 2b). This suggests that some of the Colombian women had been exposed to VAR2CSA-positive IEs during their pregnancy. These findings suggest that most of the apparently VAR2CSA-specific IgG in the Colombian plasma samples actually recognized so-far-unidentified epitopes present only in the recombinant proteins produced in insect cells. In contrast, levels of IgG reacting with FV2_BIC_, FV2_CHO_, and the corresponding native protein on IEs were positively correlated among the samples from Ghana (Fig. 2G and H and 3C), indicating that IgG recognizing these epitopes is not induced in Ghana.

### IgG reactivity to FV2_BIC_ in Colombian plasma samples cannot be depleted *in vitro* by preexposure to FV2_CHO_.

**FIG 4:**
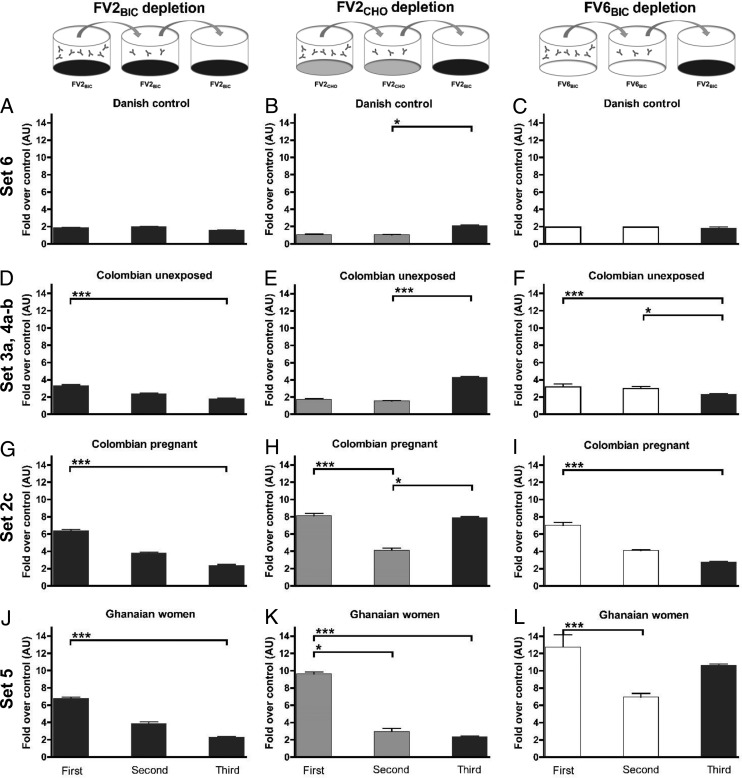
Antibody levels to recombinant PfEMP1 full-length proteins in plasma pools tested in serial ELISAs. Shown are IgG levels in pooled plasma from Danish control donors (set 6) (A to C), unexposed Colombians (sets 3a, 4a, and 4b) (D to F), Colombian pregnant women (set 2c) (G to I), and Ghanaian women (set 5) (J to L). In each of the three ELISA series (series 1 [A, D, G, and J], series 2 [B, E, H, and K], and series 3 [C, F, I, and L]), the plasma pools were transferred directly from the first to the second ELISA plates and then from the second to the third ELISA plates. In series 1, an FV2_BIC_-specific ELISA was used for all three consecutive measurements. In series 2, the first two measurements were FV2_CHO_ specific, while the third ELISA was FV2_BIC_ specific. In series 3, the first two measurements were FV6_BIC_ specific, while the third ELISA was FV2_BIC_ specific. To allow comparison of data among panels, levels are expressed as how many folds higher they were than the levels in the corresponding control ELISAs without antigen (first and second ELISAs) and shown as means and SD. *, *P* < 0.05; ***, *P* < 0.001 (using Kruskal-Wallis test followed by Dunn's multiple-comparison analysis).

We next sought to provide additional evidence that the reactivity to FV2_BIC_ among the Colombian donors was indeed directed toward non-PfEMP1-specific epitopes that are present in recombinant proteins expressed in baculovirus-transfected insect cells but absent from proteins expressed in CHO cells and absent in native PfEMP1 proteins. To this end, we sequentially measured IgG reactivity against FV2_BIC_, FV2_CHO_, and FV6_BIC_ in plasma pools transferred from one ELISA plate to the next (Fig. 4). When pools were depleted in this way of IgG reacting with FV2_BIC_ (Fig. 4A, D, G, and J), FV2_CHO_ (Fig. 4B, E, H, and K), or FV6_BIC_ (Fig. 4C, F, I, and L), respectively, reactivities in the plasma pools from Colombian (Fig. 4G to I) and Ghanaian (Fig. 4J to L) women were significantly reduced from the first ELISA to the second ELISA. In marked contrast, the reactivities to FV2_BIC_ in pools from Colombian unexposed donors (Fig. 4E) and Colombian pregnant women (Fig. 4H) were unaffected by two rounds of FV2_CHO_-specific IgG depletion, whereas essentially no reactivity was left in the Ghanaian pool treated in this way (Fig. 4K). Conversely, reactivity to FV2_BIC_ was essentially absent from each of the two initially FV2_BIC_-reactive Colombian pools after two FV6_BIC_ ELISAs (Fig. 4F and I), whereas the FV2_BIC_ reactivity in the Ghanaian pool was largely unaffected (Fig. 4L). Taken together, these depletion experiments strengthen the hypothesis that the widespread Colombian IgG reactivity to recombinant PfEMP1 antigens produced in baculovirus-transfected insect cells is not P. falciparum specific.

### Experimental exposure to P. vivax-infected mosquitoes does not induce IgG specific for VAR2CSA-type PfEMP1.

**FIG 5:**
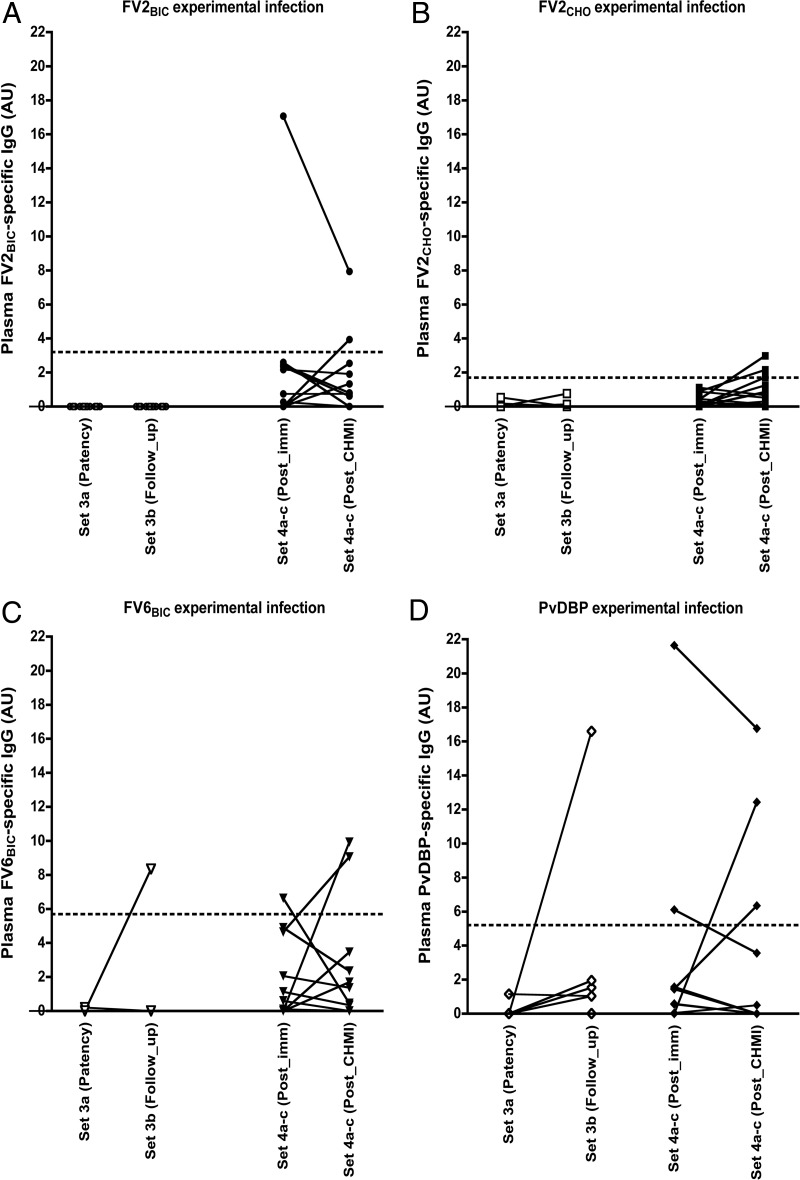
Induction of IgG responses to recombinant PfEMP1 proteins following experimental exposure to P. vivax-infected mosquitoes. Shown are plasma levels of IgG in samples from set 3 (open symbols) at the time of microscopically detectable parasitemia (patency) and 45 days after infection (follow-up) and in samples from set 4 (closed symbols) following seven rounds of immunization by infected mosquito bites (Post_imm) and 6 months after PvCHMI infection (Post_CHMI). Levels of IgG specific for FV2_BIC_ (A), FV2_CHO_ (B), FV6_BIC_ (C), and PvDBP (D) are shown. Data from individual donors are linked by lines. The panel layout is otherwise the same as in Fig. 1.

It has been proposed that exposure to P. vivax can elicit IgG responses that cross-react with VAR2CSA-type PfEMP1 (16, 23). This hypothesis is consistent with both the pregnancy restriction of VAR2CSA reactivity in Africa (where P. vivax is rare and not an important cause of placental malaria [24]) and the apparent absence of such restriction in Colombia (where P. vivax is common). Those same authors proposed that the cross-reactivity is mediated by PvDBP (17). We therefore measured levels of FV2_BIC_-, FV2_CHO_-, FV6_BIC_-, and PvDBP-specific IgG in plasma samples from 16 adults participating in a controlled human P. vivax malaria infection (PvCHMI) trial (set 3) (25). In all cases, the data were normalized by subtraction of the background reactivity at enrollment to reveal only the IgG reactivity induced in response to P. vivax infection. After normalization, no reactivity was observed at the time of patency (Fig. 5), meaning that antibodies did not develop after experimental infection. One unexposed donor showed a weak positive response to FV6_BIC_ (Fig. 5C), and one exposed donor showed a strong positive response to PvDBP (Fig. 5D) at the time of follow-up after experimental P. vivax infection. Nevertheless, several of the volunteers seroconverted for many other P. vivax antigens in response to the experimental infection (25, 26). Similar results using FV2_BIC_, FV2_CHO_, FV6_BIC_, and PvDBP were obtained with samples from 17 adult volunteers without previous malaria exposure but experimentally immunized by repeated exposure to irradiated (Duffy-positive [Fy^+^] donors [set 4a]) or nonirradiated (Duffy-negative [Fy^−^] donors [set 4c]) P. vivax-infected mosquitoes (27) (Fig. 5). Taken together, these data indicate that exposure to P. vivax parasites does not induce IgG that robustly recognizes PfEMP1 in general or VAR2CSA-type PfEMP1 in particular.

### PvDBP-specific antibodies do not recognize PfEMP1.

**FIG 6:**
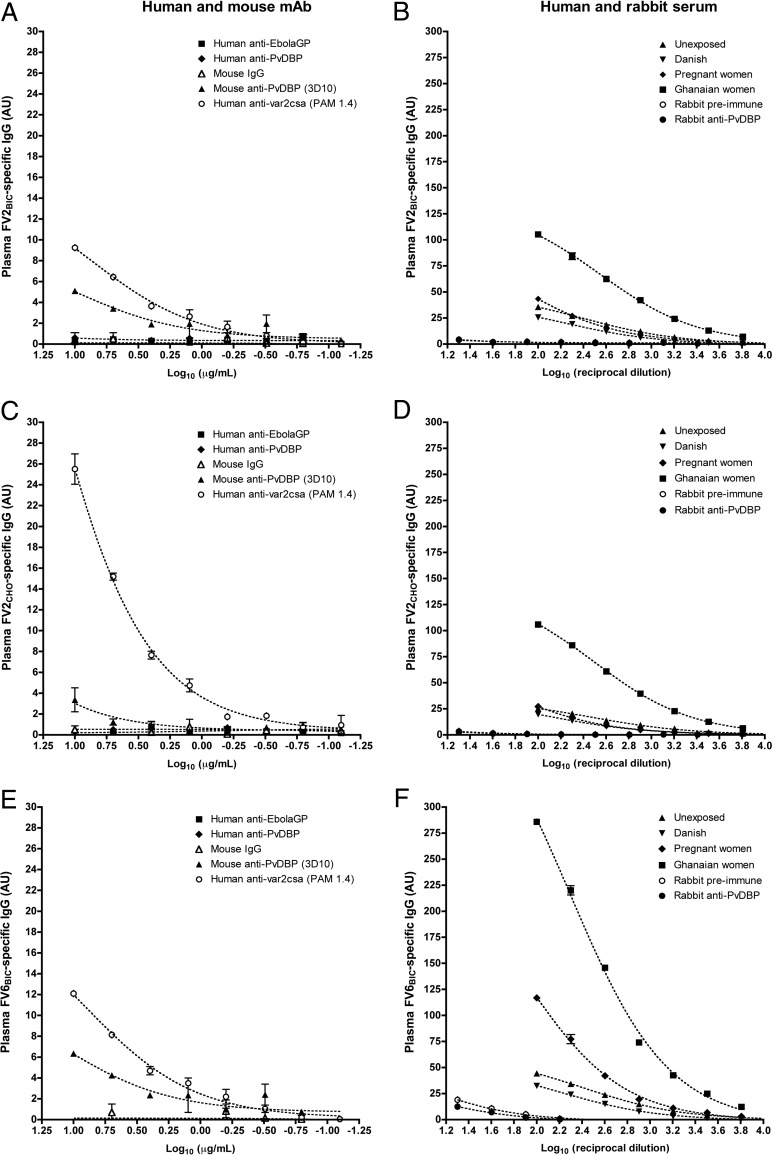
Reactivity of PvDBP-specific IgG with recombinant PfEMP1 proteins. Shown are ELISA reactivities of PvDBP-specific human (DBL10) and mouse (3D10) monoclonal antibodies (mAb) (A, C, and E) and PvDBP-specific rabbit antiserum (B, D, and F) with the recombinant full-length PfEMP1 proteins FV2_BIC_ (A and B), FV2_CHO_ (C and D), and FV6_BIC_ (E and F). (A, C, and E) Reactivities of irrelevant (Ebola virus-specific) human monoclonal IgG (EBL040), mouse polyclonal IgG, as well as the VAR2CSA-type PfEMP1-specific human monoclonal antibody PAM1.4. (B, D, and F) IgG reactivities in human plasma pools from unexposed Colombians, infected pregnant Colombians, exposed Ghanaian women, unexposed Danish adults, and preimmune rabbit serum are shown for comparison. Serial 2-fold dilution series are shown for all antibody reagents.

To investigate further whether the Colombian plasma IgG reactivity with recombinant PfEMP1 proteins could be due to cross-reactivity with IgG induced by PvDBP, we tested the ability of PvDBP-specific antibodies raised in different species to recognize recombinant and native PfEMP1. We first tested the reactivity of the mouse monoclonal antibody 3D10 (28), which was previously reported to cross-react with VAR2CSA-type PfEMP1 (17). This monoclonal antibody in fact showed low reactivity with FV2_BIC_ (endpoint titer of 0.08 μg/ml using mouse IgG as a reference) (Fig. 6A) but reacted only very weakly with FV2_CHO_ (endpoint titer of 10 μg/ml) (Fig. 6C) and not with the corresponding native PfEMP1 protein IT4VAR04 (see Fig. S1B to S1D in the supplemental material). The 3D10 antibody reacted weakly with FV6_BIC_ (Fig. 6E), much to the same extent as it reacted with FV2_BIC_ (endpoint titer of 0.08 μg/ml), but 3D10 did not label the native counterpart of FV6, HB3VAR06 (Fig. S2 B). It thus appears that 3D10, in addition to its specificity for PvDBP (28), also has some reactivity with recombinant proteins expressed in baculovirus-transfected insect cells. In marked contrast, we observed no reactivity of the PvDBP-specific human monoclonal antibody DBL10, generated from memory B cells of a volunteer in a recent PvDBP vaccination trial (29; Thomas Rawlinson, unpublished data), with any of the recombinant full-length PfEMP1 proteins (Fig. 6A, C, and E) or with IT4VAR04-positive (Fig. S1E) or HB3VAR06-positive (Fig. S2C) IEs. A corresponding lack of reactivity was observed with an antiserum from PvDBP-immunized rabbits (30) (Fig. 6B, D, and F and Fig. S1F and S2D). All the following PvDBP-specific antibody reagents showed high reactivity with a recombinant PvDBP expressed in Escherichia coli (28): rabbit anti-PvDBP serum (optical density [OD] of 0.48 ± 0.10 at a 1:2,560 dilution), DBL10 (OD of 2.88 ± 0.16 at 0.08 μg/ml), and 3D10 (OD of 1.86 ± 0.09 at 0.08 μg/ml). Taken as a whole, data from these experiments indicate that immunization with PvDBP does not generally induce antibodies that cross-react with PfEMP1.

## DISCUSSION

VAR2CSA-type PfEMP1 proteins mediate the selective accumulation of IEs infected with P. falciparum in the placenta (19, 31–33). This can have serious consequences for a pregnant woman as well as for her unborn child (fetus), because the ensuing inflammation compromises the transfer of nutrients, antibodies, and waste products between mother and fetus (reviewed in reference 7). Placental malaria is an important component of the unacceptable infection burden imposed by P. falciparum on people living in the tropics, in particular sub-Saharan Africa. More than 50 million pregnancies each year are at risk of placental malaria (34), and >100,000 infants die because of placental malaria (5). Following exposure to VAR2CSA-type PfEMP1 during pregnancy, women acquire substantial IgG-mediated protection against placental malaria, which is therefore markedly concentrated among women of low parity, in particular primigravidae (reviewed in reference 6). This fact, and the identification of the so-called minimal binding domain in VAR2CSA-type PfEMP1 that binds to its cognate receptor, oncofetal chondroitin sulfate, has raised hopes that it may be possible to vaccinate against placental malaria (35–38). Clinical phase Ia/b trials of such vaccines are currently in progress (39, 40).

A few years back, Gnidehou et al. (16) reported widespread IgG reactivity to VAR2CSA-type PfEMP1 in Colombian children and adults of both sexes and in women of all parities. Those findings are in marked contrast to essentially all other studies, mostly from Africa, documenting that high levels of VAR2CSA-specific IgG are restricted to pregnant women, that levels correlate with parity, and that VAR2CSA-specific IgG declines to low levels soon after delivery (reviewed in reference 6). Those authors speculated that the reason for their unanticipated findings might be the presence of another malaria parasite, P. vivax, which is essentially absent from most of Africa. More specifically, they recently proposed that VAR2CSA-specific IgG might be induced by cross-reactive epitopes in the P. vivax antigen PvDBP (17). The studies by Gnidehou et al. (16, 17) thus point to a completely new and unanticipated mode of acquisition and maintenance of VAR2CSA-specific IgG, although BLAST analysis of IT4VAR04 showed only low sequence homology (26%) with PvDBP (PVP01_0623800.1-p1). The findings from Colombia also raise questions regarding the rationale that underpins current efforts to develop VAR2CSA-based vaccines to protect young women against placental malaria (reviewed in reference 41). We therefore sought to examine in detail the unexpected results from the Colombian studies, using both recombinant and native VAR2CSA proteins. We used full-length recombinant proteins rather than the smaller constructs (DBL5ε, DBL3X, and ID1-ID2) used in the previous studies (16, 17), as several domains are required for optimal VAR2CSA-mediated IE sequestration (42, 43). We did not find evidence that VAR2CSA-specific IgG is acquired any differently in Colombia than in Africa. Furthermore, we found no indication to support the hypothesis that exposure to P. vivax plays a role in these processes. Instead, our data show that responses to recombinant antigens can be misleading if not checked carefully against data obtained with the corresponding native antigens. Specifically, many Colombians appear to possess IgG that reacts with recombinant PfEMP1 proteins expressed in baculovirus-transfected insect cells. Whether this is also the case for other proteins not related to malaria is not known. Importantly however, IgG reacting with a recombinant protein expressed in this way (e.g., FV2_BIC_) does not necessarily react with a corresponding protein expressed in another system (e.g., FV2_CHO_) or indeed with the native protein that it represents (e.g., IT4VAR04). A possible explanation for the discrepancy between FV2_BIC_ IgG reactivity on the one hand and FV2_CHO_ and IT4VAR04 reactivity on the other is the marked differences in protein glycosylation among malaria parasites (largely limited to the addition of glycosylphosphatidylinositol [GPI] anchors) (reviewed in references 44 and 45), insect cells (simple carbohydrates only) (reviewed in reference 46), and CHO cells (complex, human-like carbohydrates) (reviewed in reference 47). IgG reacting with recombinant antigens thus does not necessarily react (only) with the antigen-specific protein core but may also recognize posttranslational modifications that the antigen can share with many unrelated proteins produced in the same system. In the present case, plasma IgG reactivity to FV2_BIC_ among the Colombian donors seemed to be largely of the latter kind, whereas FV6_BIC_ reactivity (and FV2_BIC_ reactivity among the infected pregnant women) appeared to be a mix of both. We did not pursue the nature of the epitopes in the baculovirus proteins recognized by IgG in plasma from Colombia but not from Ghana, as this is likely to be a major undertaking that is well beyond the scope of the present work. For the same reasons, we did not investigate why these antibodies are common in Colombia while seemingly absent in Africa. It might be worth noting that the highest IgG responses to insect cell-expressed recombinant antigens among Colombian donors were found among children, both here and in the previous study. Exposure of Colombians to other pathogens or insects is another potential explanation that was not investigated here.

Apart from advising the exertion of great care when interpreting findings using recombinant proteins, we conclude from the present study that VAR2CSA-specific immunity is acquired in the same way (i.e., by exposure to VAR2CSA-positive IEs during pregnancy) in Colombia as in Africa. Furthermore, acquired immunity to VAR2CSA-type PfEMP1 seems not to be influenced by exposure to P. vivax. The rationale for the development of a VAR2CSA-based vaccine against placental malaria therefore stands (41).

## MATERIALS AND METHODS

### Ethical approval.

The original studies from which the samples used here were obtained were approved by the Institutional Review Boards of the Malaria Vaccine and Drug Development Center (MVDC) and the Centro Médico Imbanaco, Cali, Colombia; by the Institutional Review Board of the Noguchi Memorial Institute for Medical Research, University of Ghana; and by the Regional Research Ethics Committees, Capital Region of Denmark. The Colombian clinical trials were registered at ClinicalTrials.gov (registration numbers NCT01585077 and NCT01082341). The present study was approved by the Institutional Review Board at MVDC.

### Study sites.

Stored plasma samples from four previous studies involving five locations of Colombia were used. Cali, Buenaventura, Tumaco, and Quibdó are located in the western and Pacific regions, while Tierralta is situated in the northwestern Caribbean region (Table 1) (48, 49). Cali (Department of Valle del Cauca) is a city without malaria transmission. Buenaventura had an average annual parasite incidence (API) of 3.1 in the period from 2011 to 2013. It has a population of predominantly African descent, with indigenous and mestizo minorities; most malaria cases (∼75%) are caused by P. vivax. Tumaco (Department of Nariño; API = 10.3) has a highly predominant Afro-descendant population, and P. falciparum dominates (∼85%) in this region. Quibdó (Department of Chocó; API = 25) is also inhabited mainly by Afro-descendants, and most malaria episodes (∼70%) are caused by P. falciparum. Tierralta (Department of Córdoba; API = 6.7) is inhabited mostly by mestizo populations, with a small Amerindian Emberá Katío indigenous community; P. vivax dominates (∼85%) in this region.

### Study samples.

Several sets of samples were studied (Table 1). The first set (set 1) (Table 1) was obtained from a series of cross-sectional, active case detection studies carried out from 2011 to 2014 in Buenaventura, Tumaco, and Tierralta (50). Samples from children (set 1a) and men (set 1b) who were negative for malaria infection by quantitative PCR (qPCR) but with self-reported previous malaria exposure were included.

The second set of samples (set 2) was obtained from a passive case detection study conducted from 2011 to 2013 at malaria points of care (15) and from 2014 to 2016 in hospitals (51) of Quibdó, Tumaco, and Tierralta. Samples from children (set 2a) and men (set 2b) with acute P. vivax or P. falciparum malaria as well as a set of plasma samples from pregnant women with P. vivax or P. falciparum malaria (51, 52) (set 2c) were included.

The third set of samples (set 3) was collected from previously unexposed (set 3a) and semi-immune men and women (set 3b) participating in a controlled human P. vivax malaria infection (PvCHMI) trial carried out at MVDC in Cali. The unexposed (malaria antibody-negative) volunteers were from Cali, whereas the semi-immune volunteers were from Buenaventura. Their previous malaria exposure was confirmed by clinical history and by the presence of P. vivax-specific antibodies. Samples collected at enrollment, at patency (days 11 to 13), and 45 days after challenge (follow-up) were analyzed (25).

The fourth set of samples (set 4) was collected from previously unexposed adults during a clinical trial in Cali to assess the safety and protective efficacy of immunization with radiation-attenuated P. vivax sporozoites (PvRAS) (27). Samples from Duffy-positive (Fy^+^) (set 4a and set 4b) and Duffy-negative (Fy^−^) individuals (set 4c) were collected at enrollment, after seven immunizations, and after PvCHMI with nonirradiated, P. vivax-infected mosquitoes.

Plasma samples from 32 nonpregnant women from Ghana with known reactivity to VAR2CSA-type PfEMP1 (18) (set 5) and from 11 Danish adults without malaria exposure (set 6) were included as positive and negative controls, respectively.

### Recombinant parasite proteins.

The entire ectodomains of the VAR2CSA-type PfEMP1 protein IT4VAR04 (FV2_BIC_; GenBank accession number GU249598) and of the non-VAR2CSA-type PfEMP1 protein HB3VAR06 (FV6_BIC_; GenBank accession number KP_203835) were produced in baculovirus-transfected Sf9 insect cells, as described previously (20, 42). A third recombinant protein (FV2_CHO_), identical to FV2_BIC_ but expressed in suspension-adapted CHO cells (ExpiCHO; ThermoFisher Scientific) according to the manufacturer's instructions, was also used. In brief, a full-length construct with a C-terminal histidine tag was cloned into the vector pcDNA3.4 and used to transfect ExpiCHO-S cells. Glycosylation sites were not removed. Secreted recombinant proteins were purified from the supernatants by using Ni^2+^ metal chelation agarose (HisTrap HP columns; GE Healthcare).

Recombinant P. vivax Duffy binding protein region II (PvDBP), produced in Escherichia coli, was a kind gift from John H. Adams (University of South Florida) (28).

### Antibody reagents.

For the *in vitro* selection of particular PfEMP1 proteins expressed on the IE surface (see below), we used human monoclonal antibody PAM1.4, which is specific for a conformational epitope in several VAR2CSA-type PfEMP1 proteins, including IT4VAR04 (53). We used a rabbit antiserum raised against FV6_BIC_ (20) to select for IE surface expression of the non-VAR2CSA-type PfEMP1 protein HB3VAR06.

PvDBP-specific mouse monoclonal IgG1 antibody 3D10 (28) was a kind gift from John H. Adams (University of South Florida). The human monoclonal IgG1 antibody DBL10, also specific for PvDBP (our unpublished data); the human monoclonal EBL040 IgG1 antibody, specific for an Ebola virus antigen (used here as a control reagent); as well as rabbit anti-PvDBP serum (30) were kind gifts from Thomas Rawlinson and Simon Draper (University of Oxford, UK).

### Assessment of the IgG antibody response to recombinant proteins.

IgG reactivity against FV2_BIC_, FV2_CHO_, FV6_BIC_, and PvDBP was measured in duplicate by an ELISA as described previously (18, 28, 54). Briefly, 96-well flat-bottom microtiter plates (Nunc MaxiSorp) were coated with recombinant protein in Dulbecco's phosphate-buffered saline (Lonza). After blocking and washing, human plasma samples (1:400), human and mouse monoclonal antibodies (0.08 to 10 μg/ml), or rabbit serum (1:20 to 1:2,560) was added, followed by horseradish peroxidase-conjugated rabbit anti-human IgG (1:3,000; Dako), goat anti-mouse IgG (1:1,000; ThermoFisher), or goat anti-rabbit IgG (1:3,000; Dako). Bound antibodies were detected by adding tetramethylbenzidine (TMB PLUS2; Eco-Tek), and the reaction was stopped by the addition of 0.2 M H_2_SO_4_ to the mixture. The optical density (OD) was read at 450 nm, and the specific antibody levels were calculated in arbitrary units (AU), as described previously (18). Negative cutoff values were calculated as the mean AU values plus 2 standard deviations (SD) obtained with the Danish control samples (set 6) (Table 1).

### Depletion of IgG reactivity to recombinant proteins.

Pooled plasma samples from Colombian pregnant women (set 2c; *n* = 56) and adults without previous malaria exposure (sets 3a, 4a, and 4b; *n* = 22 enrollment samples), malaria-exposed nonpregnant women from Ghana (set 5; *n* = 32), and unexposed Danish adults (set 6; *n* = 11) were tested in three sequential ELISAs. In the first ELISA, levels of IgG specific for FV2_BIC_, FV2_CHO_, and FV6_BIC_ were measured. After incubation (1 h), the depleted pools were transferred directly to new plates coated with the same protein used in the first ELISA. After incubation (1 h), the further depleted pools were transferred to FV2_BIC_-coated plates and once again incubated as described above. Further processing was performed as described above. For these experiments, the data were normalized by dividing the sample OD by the OD for the corresponding negative control (fold over control).

### Malaria parasite culture and selection *in vitro*.

P. falciparum clones IT4/FCR3 and HB3 were maintained in serum-free medium as described previously (55). Erythrocytes infected by late-stage IT4/FCR3 parasites were selected for surface expression of IT4VAR04 by immunomagnetic selection using protein A-coupled DynaBeads coated with PAM1.4, as described previously (56). A similar approach was used to select HB3-IEs for surface expression of HB3VAR06, using an HB3VAR06-specific rabbit antiserum (20). Transcription of the relevant *var* genes and IE surface expression of the corresponding PfEMP1 protein were monitored by quantitative real-time PCR (57) and flow cytometry (56), respectively. The genotypic identity of the parasites (58) and the absence of Mycoplasma contamination determined by using the MycoAlert mycoplasma detection kit (Lonza) were verified regularly.

### Antibody reactivity with the surface of P. falciparum-infected erythrocytes.

Plasma IgG reactivity against IEs expressing IT4VAR04 or HB3VAR06 was analyzed by flow cytometry, as described previously (56), using a Beckman Coulter FC500 flow cytometer for data acquisition and FlowLogic software (Inivai Technologies, Australia) for list mode data analysis. IgG binding to IEs was quantified as the relative median fluorescence intensity (rMFI) using the equation 100 × [(MFI_sample_ − MFI_blank_)/(MFI_positive control_ − MFI_blank_)]. Negative cutoff values were calculated as the mean of rMFI values plus 2 SD obtained with the Danish control samples (set 6).

### Statistical analysis.

Data were analyzed by using GraphPad Prism version 6.0 (GraphPad Software, San Diego, CA, USA). All experiments reported here were repeated at least twice, with similar results. The Mann-Whitney U or Kruskal-Wallis test followed by Dunn's multiple-comparison test was used to compare two or more than two groups, respectively. Spearman's rank correlation (*r_s_*) was used to assess the correlation between numeric variables. A chi-square test was used to compare differences in proportions. *P* values of <0.05 were considered statistically significant.

## Supplementary Material

Supplemental material
