# Expression of Concern for Lopez-Perez et al., “IgG Responses to the *Plasmodium falciparum* Antigen VAR2CSA in Colombia Are Restricted to Pregnancy and Are Not Induced by Exposure to *Plasmodium vivax*”

**DOI:** 10.1128/iai.00545-24

**Published:** 2025-01-13

**Authors:** American Society for Microbiology

## EXPRESSION OF CONCERN

The American Society for Microbiology (ASM) and *Infection and Immunity* (IAI) are issuing this Expression of Concern regarding the following publication:

Lopez-Perez M, Larsen MD, Bayarri-Olmos R, Ampomah P, Stevenson L, Arévalo-Herrera M, Herrera S, Hviid L. 2018. IgG responses to the *Plasmodium falciparum* antigen VAR2CSA in Colombia are restricted to pregnancy and are not induced by exposure to *Plasmodium vivax*. Infect Immun 86:10.1128/iai.00136–18. https://doi.org/10.1128/iai.00136-18.

ASM and IAI were notified of serious concerns regarding this study, including potential mishandling of human samples and animals involved in the study and improper execution of study protocols. The authors have not provided appropriate information regarding any potential ethical approvals they received from the institutional review boards of the Malaria Vaccine and Drug Development Center (MVDC) and the Centro Médico Imbanaco, Cali, Colombia.

Additionally, Colombian authorities have apprehended 180 rodents who did not receive the necessary maintenance and living conditions (J. Roa, 2023, https://www.cali.gov.co/seguridad/publicaciones/175100/autoridades-realizaron-aprehension-preventiva-de-180-especimenes-en-laboratorio-de-caucaseco/) and ultimately closed down the facility (M. Wadman, Science, 2023, https://doi.org/10.1126/science.adi4928). Subsequently, the NIH defunded Caucaseco Scientific Research Center and removed it from its list of approved animal facilities (P. Jacobs, Science, 2023, https://doi.org/10.1126/science.adj6184).

ASM has formally requested that this paper and these concerns be reviewed as part of an ongoing “scientific misconduct investigation” by the Universidad del Valle and are awaiting their response. ASM is following its Publishing Ethics Policies and Procedures by publishing this Expression of Concern to raise awareness of the above-mentioned concerns; it will be updated accordingly pending the outcome of the investigation.

