# Supplementary material for: IgG Responses to the Plasmodium falciparum Antigen VAR2CSA in Colombia Are Restricted to Pregnancy and Are Not Induced by Exposure to Plasmodium vivax
Source: Infect Immun. 2018 Jul 23;86(8):e00136-18. doi: 10.1128/IAI.00136-18 (PMC6056870; doi:10.1128/IAI.00136-18)
Supplement: Supplemental material [file supp_86_8_e00136-18__index.html]

Supplemental material 

# IgG Responses to the Plasmodium falciparum Antigen VAR2CSA in Colombia Are Restricted to Pregnancy and Are Not Induced by Exposure to Plasmodium vivax

## Supplemental material

- Supplemental file 1 -

  Fig. S1. Reactivity of antibody reagents with *P. falciparum* IEs expressing IT4VAR04 on the surface. Fig. S2. Reactivity of antibody reagents with *P. falciparum* IEs selected to express HB3VAR06 on the surface. Fig. S3. Correlation of antibody responses to native and recombinant VAR2CSA-type PfEMP1.

  PDF, 494K
