# Supplementary material for: IgG Responses to the Plasmodium falciparum Antigen VAR2CSA in Colombia Are Restricted to Pregnancy and Are Not Induced by Exposure to Plasmodium vivax
Source: Infect Immun. 2018 Jul 23;86(8):e00136-18. doi: 10.1128/IAI.00136-18 (PMC6056870; doi:10.1128/IAI.00136-18)

***Supplementary Figure 1. Reactivity of antibody reagents with* P. falciparum *IEs expressing IT4VAR04 on the surface***

IgG reactivity of IT4VAR04-positive IEs with pools of human plasma (1:20) from Danish control donors (gray), malaria-unexposed Colombians (red), malaria-infected Colombian pregnant women (green), and malaria-exposed Ghanaian women with one or more previous pregnancies (blue) (**A**). Reactivity of IT4VAR04-positive IEs with the PvDBP-specific mouse monoclonal antibody 3D10 (10 µg/mL) (**B**, **C**), an unspecific mouse IgG antibody (**D**), the *Pv*DBP-specific human monoclonal antibody DBL10 (10 µg/mL) (**E**), and a rabbit *Pv*DBP-specific serum (**F**).

***Supplementary Figure 2. Reactivity of antibody reagents with* P. falciparum *IEs selected to express HB3VAR06 on the surface***

IgG reactivity of HB3VAR06-positive IEs with pools of human plasma from Danish control donors (gray), malaria-unexposed Colombians (red), malaria-infected Colombian pregnant women (green), and malaria-exposed Ghanaian women with one or more previous pregnancies (blue) (**A**). Reactivity of HB3VAR06-positive IEs with the PvDBP-specific mouse monoclonal antibody 3D10 (**B**), the PvDBP-specific human monoclonal antibody DBL10 (**C**), and a rabbit PvDBP-specific serum (**D**).

***Supplementary Figure 3. Correlation of antibody responses to native and recombinant VAR2CSA-type PfEMP1***

Correlation ($r_s$) of IgG levels against recombinant ($FV2_{CHO}$; determined by ELISA) and corresponding native (IT4VAR04; determined by flow cytometry) VAR2CSA-type PfEMP1 proteins in individual samples from Set 1a/2a (**A**), 1b/2b (**B**), and 2c (**C**). Panel layout as in Fig. 3D.

*Supplementary Fig. 1*

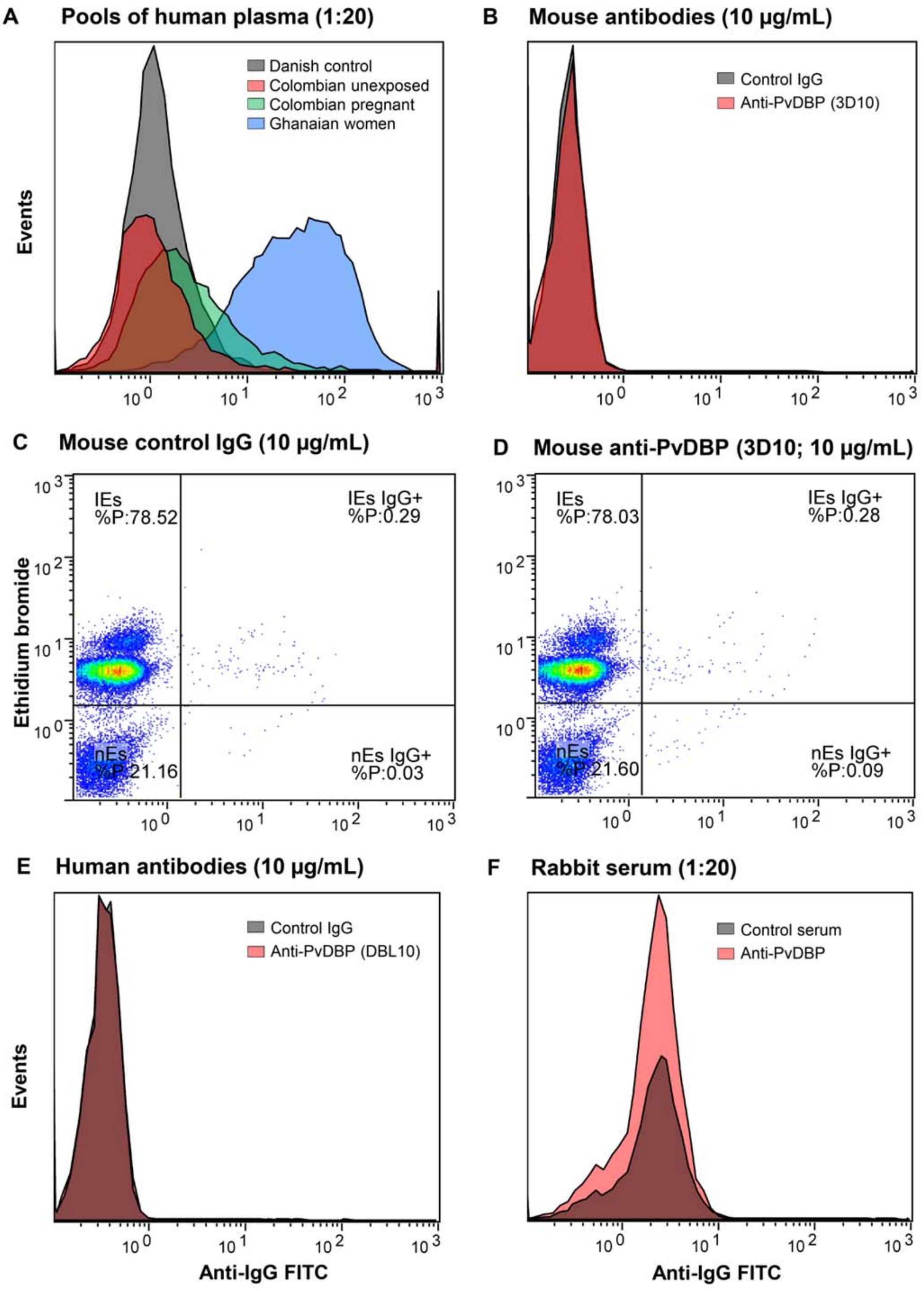

*Supplementary Fig. 2*

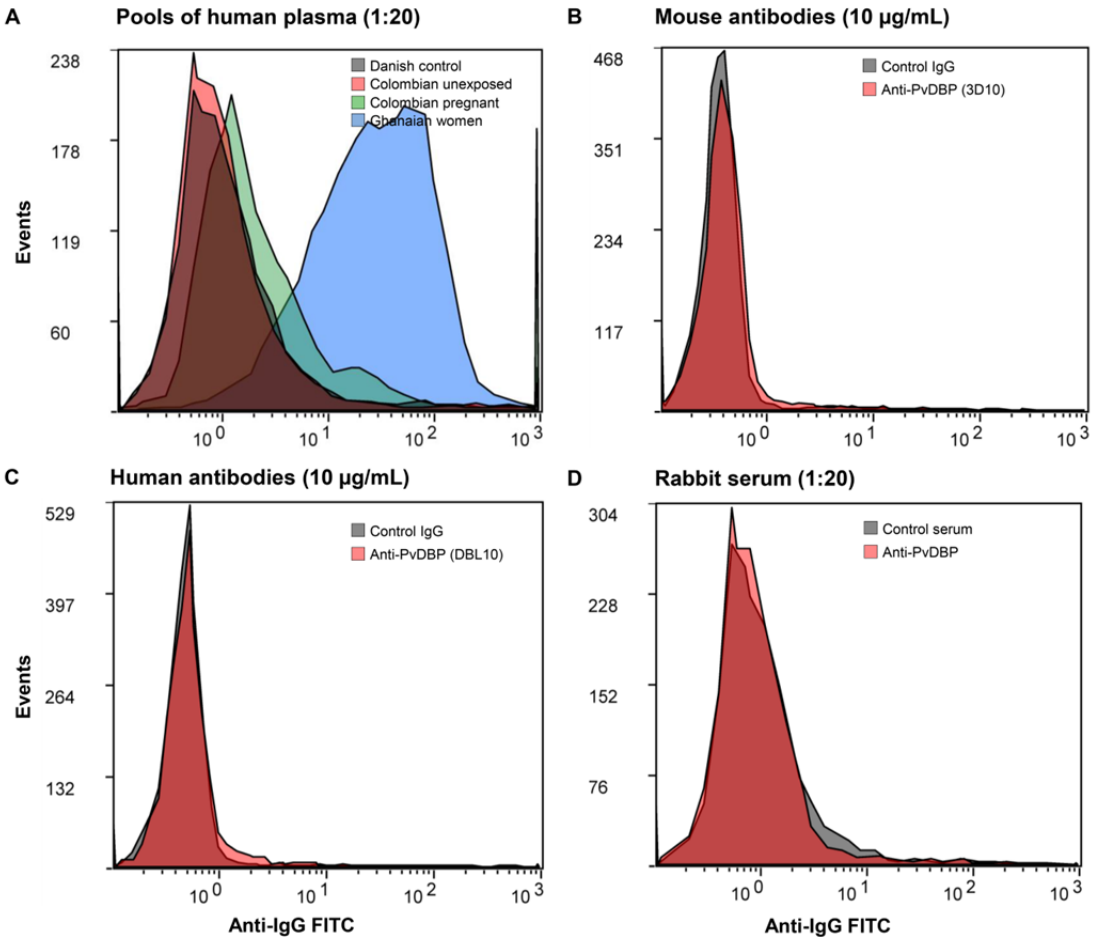

***Supplementary Fig. 3***

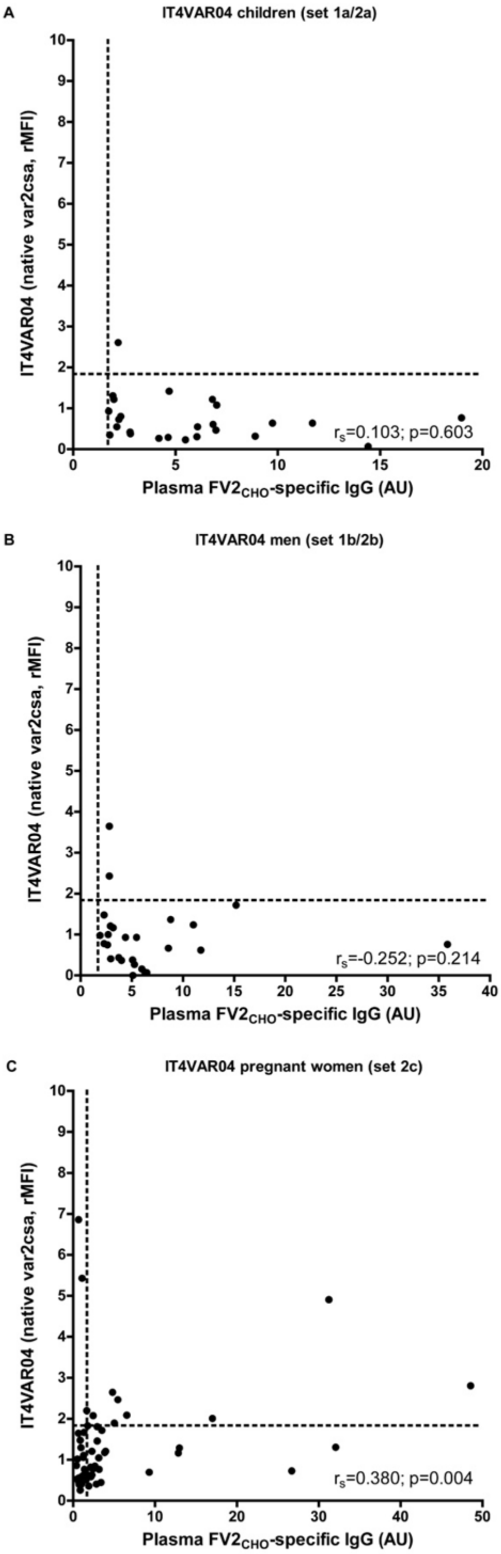

Supplement: Supplemental material [file IAI.00136-18_zii999092477s1.pdf]
